# Secretion of Soluble Vascular Endothelial Growth Factor Receptor 1 (sVEGFR1/sFlt1) Requires Arf1, Arf6, and Rab11 GTPases

**DOI:** 10.1371/journal.pone.0044572

**Published:** 2012-09-04

**Authors:** Jae-Joon Jung, Ajit Tiwari, Shivangi M. Inamdar, Christie P. Thomas, Apollina Goel, Amit Choudhury

**Affiliations:** 1 Departments of Anatomy and Cell Biology, University of Iowa, Iowa City, Iowa, United States of America; 2 Departments of Orthopaedics and Rehabilitation, University of Iowa, Iowa City, Iowa, United States of America; 3 Department of Internal Medicine, University of Iowa, Iowa City, Iowa, United States of America; 4 Department of Radiation Oncology, University of Iowa, Iowa City, Iowa, United States of America; University of Nebraska Medical Center, United States of America

## Abstract

The soluble form of vascular endothelial growth factor receptor 1 (sVEGFR-1/sFlt1) is generated by alternative splicing of the FLT1 gene. Secretion of sFlt1 from endothelial cells plays an important role in blood vessel sprouting and morphogenesis. However, excess sFlt1 secretion is associated with diseases such as preeclampsia and chronic kidney disease. To date, the secretory transport process involved in the secretion of sFlt1 is poorly understood. In the present study, we investigated the itinerary of sFlt1 trafficking along the secretory pathway. To understand the timecourse of sFlt1 secretion, endothelial cells stably expressing sFlt1 were metabolically radiolabeled with [^35^S]-methionine and cysteine. Our results indicate that after initial synthesis the levels of secreted [^35^S]-sFlt1 in the extracellular medium peaks at 8 hours. Treatment with brefeldin A (BFA), a drug which blocks trafficking between the endoplasmic reticulum (ER) and the Golgi complex, inhibited extracellular release of sFlt1 suggesting that ER to Golgi and intra-Golgi trafficking of sFlt1 are essential for its secretion. Furthermore, we show that ectopic expression of dominant-negative mutant forms of Arf1, Arf6, and Rab11 as well as siRNA-mediated knockdown of these GTPases block secretion of sFlt1 during normoxic and hypoxic conditions suggesting role for these small GTPases. This work is the first to report role of regulatory proteins involved in sFlt1 trafficking along the secretory pathway and may provide insights and new molecular targets for the modulation of sFlt-1 release during physiological and pathological conditions.

## Introduction

Soluble VEGFR-1 (sFlt1) is generated by alternative splicing of the *FLT1* gene [1]. sFlt1 binds to all isoforms of VEGF-A and placenta growth factor (PlGF) with high affinity [1], [2]. It can trap both VEGF-A and PlGF and inhibit their biological activities and can also form VEGF-stabilized complex with the extracellular domain of VEGFR2 [1], [3]. In pregnancies complicated with preeclampsia, sFlt1 is secreted at high levels [4], [5]. Maternal serum levels of sFlt1 are elevated five weeks prior to the onset of preeclampsia, supporting the premise that sFlt1 is a key factor responsible for the clinical manifestation of this disorder [4]. An increased sFlt1 level is associated with endothelial dysfunction in chronic kidney disease and correlates well with the prediction of cardiovascular risk associated with this disease [6]. In physiological settings, sFlt1 bind and sequester VEGF-A at a distance from the endothelial cell surface for proper vessel morphogenesis [7].

Constitutive membrane transport is fundamental to a number of cellular functions including growth and differentiation, secretion of proteins, as well as generation, homeostasis, and turnover of cellular organelles. Such transport processes are coordinated by internal regulators that ensure fidelity and uninterrupted flow. Certain basic steps in membrane and protein transport such as vesicle formation, docking, and fusion have been studied extensively, and a fundamental set of molecular mechanisms executing these events has been elucidated. Membrane trafficking regulatory molecules such as Rab GTPases, Arf GTPases, and soluble N-ethylmaleimide-sensitive factor attachment protein receptor (SNARE) family of proteins have been implicated in secretion of souble cargo molecules [8], [9], [10], [11], [12]. Superimposed on this core machinery, regulatory mechanisms must exist to fine tune membrane traffic, maintain organelle homeostasis, and mediate adaptive responses of transport to variations in extracellular conditions. In spite of their potentially great physiologic and pathologic importance of sFlt1 secretion, and intracellular transport mechanisms has received less attention.

In the current study, we investigated the mechanism of secretory transport leading to extracellular secretion of sFlt1. In mammalian cells, the Golgi apparatus is a central hub for membrane trafficking, which receives newly synthesized proteins and lipids from the endoplasmic reticulum (ER), modifies many of the proteins, and sorts them to various destinations [13], [14], [15], [16]. We show that newly synthesized sFlt1 is transported from ER to Golgi complex and subsequently transported via post-Golgi trafficking mechanisms and finally secreted extracellularly. Next, we hypothesized that certain SNAREs, Rab- and Arf-GTPases may regulate the secretion of sFlt1 because these proteins play key roles in the regulation of intracellular vesicular trafficking cargo molecules [8], [9], [10], [11], [12]. Our results show for the first time role for Rab11, Arf1, and Arf6 GTPases in secretion of the sFlt1 during normoxic and hypoxic culture conditions.

## Results

### Soluble VEGFR1/sFlt1 expression and secretion in endothelial cell

**Figure 1:**
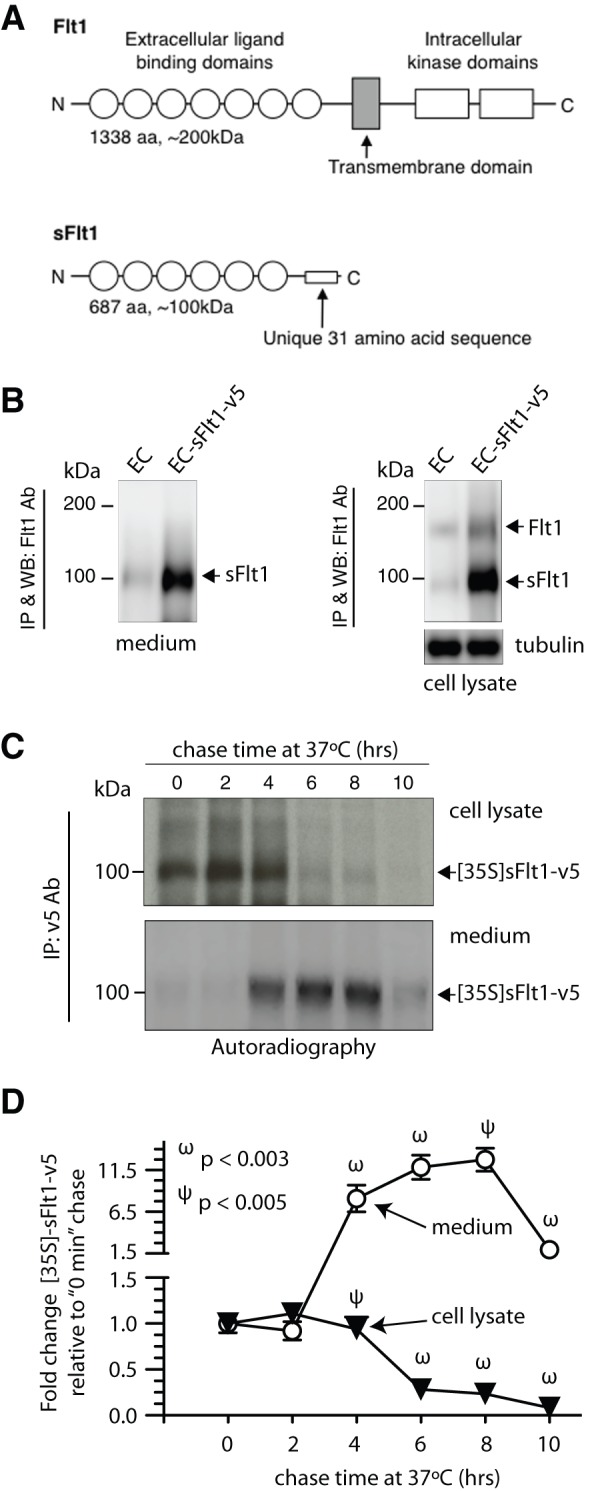
*A*: Schematic representation of domain organization of full-length Flt1 and soluble Flt1 (sFlt1). ***B***: HUVECs stably expressing v5-tagged sFlt1 (EC-sFlt1) and HUVEC control cells were seeded at 30% confluency and cultured for 5 days before collection of spent culture media and preparation of cell lysates. Immunoprecipitation (IP) of endogenous sFlt1 and ectopic sFlt1-v5 from cell lysates and spent culture supernatants was performed using an Ab to Flt1. Blot shows robust cellular expression and secretion of sFlt1-v5 in EC-sFlt1 stable cells relative to control HUVECs (EC). ***C***: Time course of sFlt1-v5 secretion after synthesis. EC-sFlt1 cells (80% confluency) were metabolically labeled with ^35^S-methionine-^35^S-cysteine for 20 min. After the indicated chase period in an excess of unlabeled methionine-cysteine, cells were lysed, and spent culture media and cell lysates were TCA precipitated. Radioactive counts were normalized prior to IP using an anti-v5 Ab. The immunoprecipitated samples were analyzed by SDS-PAGE; gels were dried and exposed for autoradiography. ***D***: Autoradiographic signals from films were measured using Image J (NIH) software and are presented as fold change relative to the 0 min chase values. Values are mean ± SEM; n = 3.

The full-length Flt1 coding RNA contains 30 spliced exons and is translated into a ∼200 kDa transmembrane protein with an extracellular N-terminal ligand-binding domain, a single membrane-spanning segment, and a C-terminal intracellular segment that carries two tyrosine kinase domains. Soluble VEGFR-1/sFlt1 shares the ligand-binding domain (the first 13 exons, 687aa) with full-length VEGFR-1/Flt1, but lacks the membrane-spanning and C-terminal domains (Figure 1A) [17]. In addition to sFlt1, other soluble forms have also been reported [18], [19]. The theoretical molecular weight of sFlt1 is around 78 kDa, but the experimental size range is 85–120 kDa. As a consequence, sFlt1 is secreted as a ∼100 kDa protein, which can bind VEGF and PLGF with high affinity and function as a circulating VEGF and PLGF antagonist.

sFlt1 was first identified in HUVEC conditioned media [17]. As expected, we also observed two Flt1 antibody (Ab)-reactive weak bands corresponding to full-length Flt1 (180 kDa) and sFlt1 (100 kDa) in cell lysates, and a weak sFlt1 band in spent culture medium (Figure 1B). Since pathologic conditions such as hypoxia result in up-regulated secretion of sFlt1, we generated stable HUVEC cell lines expressing enhanced levels of v5-tagged sFlt1 (EC-sFlt1, Figure 1B). To determine the duration of sFlt1 secretion after synthesis, we pulse-labeled EC-sFlt1 cells with ^35^S-Met-Cys for 20 min and then chased for various times. We detected newly synthesized [^35^S]-sFlt1-v5 up to 4 hrs in cell lysates and in conditioned media as early as four hrs post-labeling; the level of secreted sFlt1 was maximal eight hrs post-labeling (Figure 1C, D).

### Brefeldin A blocks sFlt1 secretion

**Figure 2:**
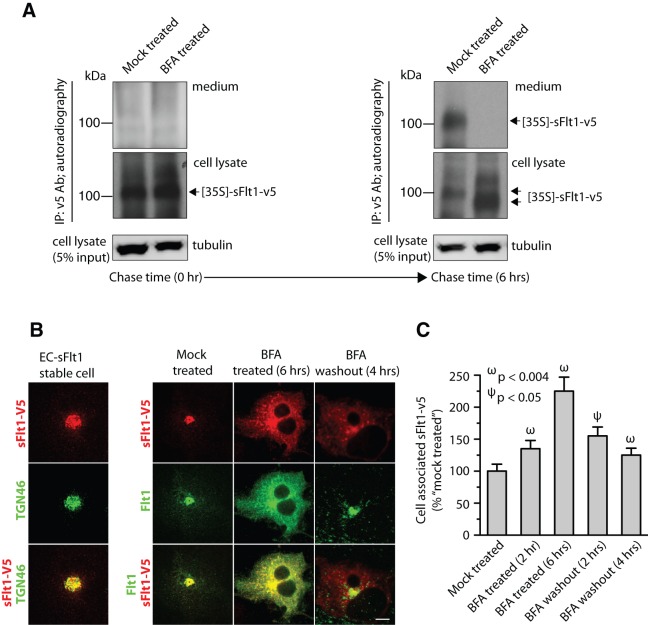
Brefeldin A (BFA) treatment blocks sFlt1 secretion and increases intracellular accumulation. ***A***: EC-sFlt1 cells were either mock treated or pretreated with BFA (1 μg/ml) for 20 min before metabolically labeling with ^35^S-methionine-^35^S-cysteine for 20 min. Samples were then chased for 6 hrs in excess of unlabeled methionine-cysteine containing medium without (in control) or with BFA (1 μg/ml). Cells were then lysed and spent culture supernatant and cell lysates were TCA precipitated. Radioactive counts were normalized prior to immunoprecipitation with anti-v5 Ab. The immunoprecipitated samples were analyzed by SDS-PAGE, gels were dried and exposed for autoradiography, and representative autoradiographs are shown. ***B***: EC-sFlt1 stable cells were grown in medium without (mock treated) or with BFA (1 μg/ml) for either 2 hrs or 6 hrs. A set of samples, after 6 h of BFA treatment, was subjected to BFA washout for 2 hrs or 4 hrs. Cells were fixed, permeabilized, and labeled with Abs against v5, Flt1, or TGN46 (Golgi marker) followed by the appropriate fluorescently-tagged secondary Ab. Representative images obtained by epifluorescence microscopy show localization of v5-tagged sFlt1. ***C***: Quantification of intracellular retention of v5-sFlt1 upon BFA treatment and BFA washout steps. Epifluorescence images were acquired and total cell-associated fluorescence was quantified by image analysis. Values represent relative change in the levels of v5-sFlt1 normalized to an arbitrary value of 100% for untreated controls. Results are expressed as mean ± SEM (n = 70 cells for each condition, from 3 separate experiments). Scale bar represents 5 µm.

We next investigated whether sFlt1 traffics through the Golgi complex and if this transit is essential for its secretion into the extracellular medium. We pretreated EC-sFlt1 cells for 30 min with BFA, a drug that causes resorption of the Golgi complex into the ER and inhibition of ER to Golgi protein transport [20], [21]. BFA pretreatment did not affect new synthesis of [^35^S]-sFlt1-v5 (Figure 2A). After a 6 hr chase in the presence of BFA following ^35^S-Met/Cys pulse labeling, [^35^S]-sFlt1-v5 secretion into the media was blocked with increased accumulation in cell lysates relative to control (Figure 2A) indicating that the secretion of sFlt1 depends on the vesicular transport through the ER and Golgi complex. Next, we investigated the intracellular distribution of sFlt1-v5 upon BFA treatment using immunofluorescence imaging of EC-sFlt1 cells using an Ab to the v5 tag. Control samples indicated Golgi localization of sFlt1 as Abs to TGN46 and sFlt1-v5 co-localized (Fig 2B). Increased intracellular accumulation of sFlt1-v5 in punctate vesicular structures was observed in BFA treated samples. Quantitatively, a greater than 2-fold increase in intracellular accumulation of sFlt1 was observed after BFA treatment (Figure 2C). After BFA was removed from the media, intracellular retention of sFlt1 was decreased (Figure 2B,C). These results suggest that disrupting membrane transport from the ER to the Golgi complex blocks the secretion of sFlt1 and results in increased intracellular levels of sFlt1.

### Role of Arf1, Rab11, and Arf6 in secretory transport of sFlt1

Since it is likely that sFlt1 transport along the secretory pathway occurs by vesicular trafficking, we explored the role of proteins, which regulate membrane trafficking such as Arf GTPases, Rab GTPases, and syntaxins (t-SNARE proteins) in sFlt1 secretion. EC-sFlt1 cells were transfected via nucleofection using Amaxa nucleoporator with plasmid constructs expressing dominant-negative forms of Arf, Rab GTPase family members, syntaxins, or siRNAs against Arf1, Arf6, or Rab11. With this approach, we routinely achieve >75% transfection efficiency (Figure S1A). The extent of knockdown of endogenous Arf1, Arf6, and Rab11 with siRNAs was in the range between 50–75% (Figure S2B). The levels of cellular *vs.* secreted sFlt1 protein was assessed 48 hrs post-transfection. Intracellular localization and the extent of protein accumulation were also determined by quantitative epifluorescence microscopy.

**Figure 3:**
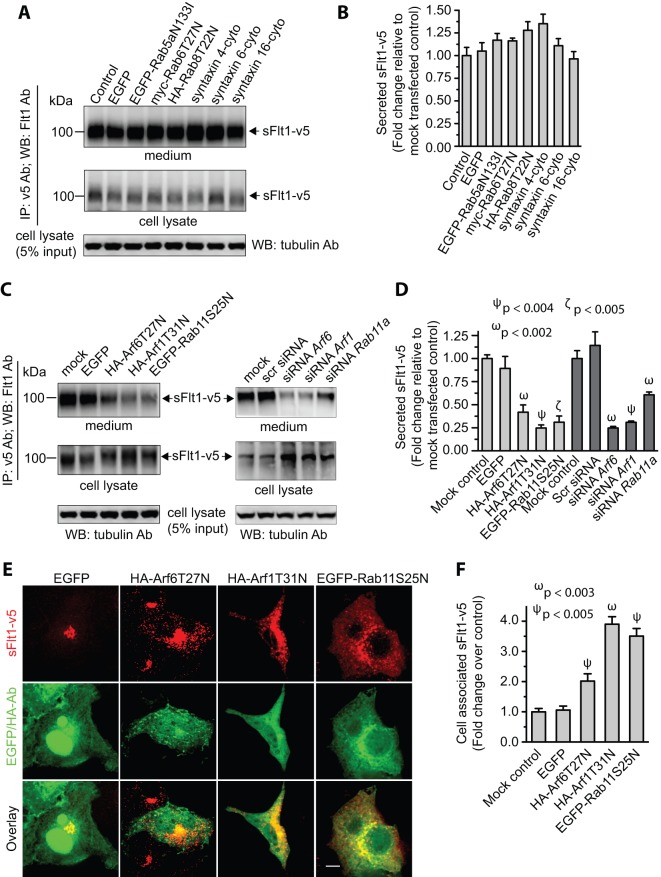
Secretion of sFlt1 from endothelial cells requires functional Arf6, Arf1, and Rab11 GTPases. ***A–F***: EC-sFlt1 cells were nucleofected with plasmid constructs expressing dominant-negative forms of Arf1, Arf6, Rab5, Rab6, Rab 8, syntaxin 4, syntaxin 6, and syntaxin 16. Similarly, in a separate study EC-sFlt1 cells were nucleofected with scramble oligonucleotide or siRNAs against *Arf1*, *Arf6*, and *Rab11a*. Media containing nucleofection complexes were replaced with fresh media 8 hrs post-nucleofection. At 36 hrs post-nucleofection, secreted and intracellular sFlt1 were immunoprecipitated from spent culture media and cell lysates using the anti-v5 Ab. (***A***, ***C***) Representative blot shows relative expression of sFlt1-v5 in cell lysates and secreted in culture media. ***B*** **,** ***D***: Band densities from experiments as in ***A, C*** were quantified by densitometric analysis. Secretion of sFlt1-v5 was calculated as the ratio of desitometric band intensity values of secreted to intracellular Flt1; this ratio was set at 1 for mock-transfected control EC-sFlt1 cells. The data are the means ± SEM from three independent experiments. ***E***: EC-sFlt1 cells were transiently transfected with indicated dominant-negative plasmid constructs. After 24 hrs cells were fixed, permeabilized, and labeled with anti-v5 Ab to detect cell-associated sFlt1-v5. Representative images obtained by epifluorescence microscopy show localization of sFlt1-v5. ***F***: Quantification of intracellular retention of sFlt1-v5 upon inhibition of Arf1, Arf6, and Rab11 function. Total cell-associated fluorescence was quantified by analysis of epifluorescence images obtained as in ***E***. Values represent relative change in the levels of sFlt1-v5 normalized to an arbitrary value of 100% for mock-transfected control. Results are expressed as mean ± SEM (n = 70 cells for each condition, from 3 separate experiments). Scale bar represents 5 µm.

Arf1 is primarily localized to the Golgi complex where it regulates the binding of cytosolic coat proteins, (such as coat protein I (COPI), adaptor protein 1, and Golgi-localized, γ-ear–containing, Arf-binding proteins (GGA)) to Golgi membranes [12], [22] and thus serves to regulate membrane traffic in the ER-Golgi system [23]. A dominant-negative form of Arf1 (Arf1T31N), which is defective in GTP-binding potently, inhibits ER to Golgi and intra-Golgi transport of vesicular stomatitis virus glycoprotein (VSV-G) [24]. Our results demonstrate that expression of this mutant reduced secretion of sFlt1 4-fold (Figure 3C,D) and resulted in an over 3-fold increase in intracellular accumulation of sFlt1 in vesicular structures (Figure 3E,F). Futhermore, siRNA mediated knockdown of endogenous Arf1 decreased secretion of sFlt1 by ∼4-fold relative to control (Figure 3C,D). These results suggest that functional Arf1 is required for sFlt1 to transit from the ER to Golgi, a step that is essential for its subsequent secretion.

Rab GTPases (such as Rab6, Rab8, and Rab11) are important regulators of vesicular traffic in the secretory pathway and are known to be involved in the transport of cargo proteins. Members of the Rab6 family are found in both the Golgi as well as cytoplasmic vesicles and they regulate protein transport between Golgi, ER, PM, and endosomes [25]. Rab8 and Rab11 are both known to be involved in trafficking between the trans-Golgi network and the plasma membrane [26], [27] ^,^ [28], [29]. In order to test their role in sFlt1 secretion, we over-expressed dominant-negative forms of Rab6, Rab8 and Rab11 in EC-sFlt1 cells; as a control, we also expressed a dominant-negative form of Rab5 which are known to be involved in homotypic fusion of clathrin-derived endocytic vesicles [30]. Among the mutants tested, only Rab11 showed an effect with an approximately 2-fold reduction in sFlt1 secretion relative to mock transfected and EGFP expressing cells (Figure 3). Increased intracellular accumulation (>3-fold) of sFlt1 was evident in cells expressing dominant-negative Rab11 (Figure 3C,D). In addition, reduction of Rab11 using a siRNA approach, decreased secretion of sFlt1 by ∼2-fold (Figure 3C,D). These results suggest that post-Golgi transport and delivery of sFlt1 to the plasma membrane requires functional Rab11.

Arf6 is member of the Arf family of GTPases found exclusively at the PM and endosome membranes; it exerts direct control over the exocytotic machinery and regulates insulin-regulated secretion and exocytosis in neuroendocrine cells [31], [32], [33], [34]. Expression of a dominant-negative form of Arf6 (Arf6T27N) known to block its function, resulted in a 2-fold reduction in sFlt1-v5 secretion in EC-sFlt1 cells (Figure 3A,B) and an increase in intracellular accumulation of sFlt1-v5 relative to control (Figure 3B,C). Similarly, reduction of endogenous Arf6 by siRNA decreased secretion of sFlt1 by ∼4-fold (Figure 3C,D). These results suggest that Arf6 GTPase also plays a role in the secretion of sFlt1.

Syntaxins are members of the t-SNARE family of proteins. They are involved in the vesicle fusion process and regulate cargo delivery from donor vesicles to the target membrane [35], [36]. We studied the role of syntaxin 4 and syntaxin 6 in sFlt1 secretion as these SNAREs are known to be involved in the secretion of cargo molecules. Syntaxin 4 participates in exocytic vesicle fusion with the plasma membrane while syntaxin 6 regulates intra-Golgi or post-Golgi fusion events [37], [38], [39], [40]. We over-expressed dominant-negative forms of syntaxin 4 or syntaxin 6 along with dominant-negative syntaxin 16 as a control. (Syntaxin 16 is known to be exclusively involved in the retrograde transport from endosomes to the Golgi complex [41]). We found that inhibition of syntaxin 4, syntaxin 6, or syntaxin 16 did not alter sFlt1 secretion (Figure 3A,B).

### The role of Arf1, Arf6, and Rab11 on hypoxia-induced secretion of sFlt1 from JEG3 cells

Hypoxia is known to increase the expression and secretion of sFlt1 in cytotrophoblasts and trophoblasts [42]. Placental hypoxia increases the risk of stillbirth and pregnancy-related diseases, such as preeclampsia [42]. Our results suggest that Arf1, Arf6, and Rab11 GTPases are involved in the secretion of sFlt1 in endothelial cells. Therefore, we investigated role of these GTPases in hypoxia-induced secretion of sFlt1 from JEG3 cells; these cells have features of both cytotrophoblasts and extravillous trophoblasts [19].

**Figure 4:**
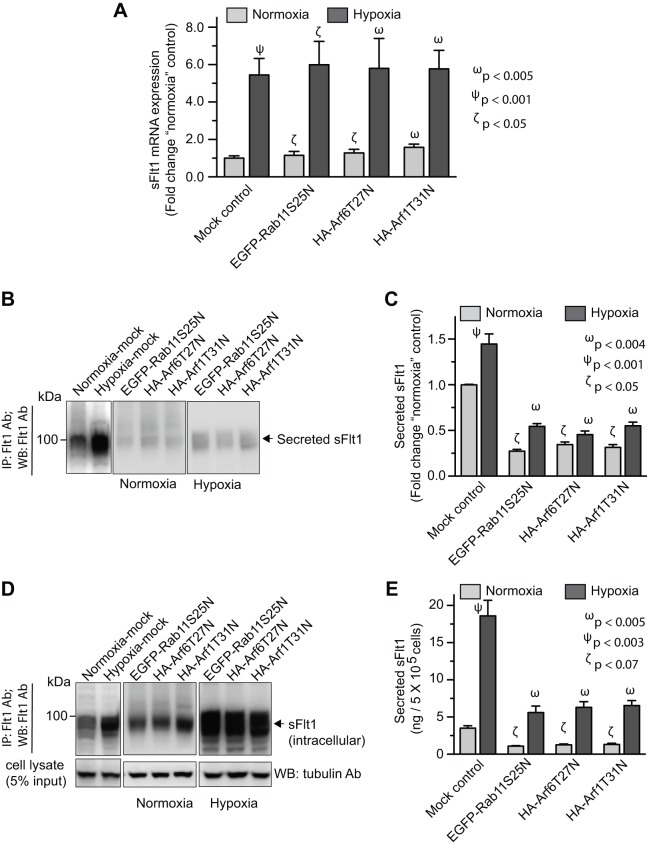
Role of Rab11, Arf1, and Arf6 GTPases in the secretion of sFlt1 in JEG3 cells during normoxic and hypoxic culture conditions. JEG-3 cells were nucleofected with plasmid constructs expressing dominant negative forms of Arf1 (HA-tagged-Arf1T31N), Arf6 (HA-tagged-Arf6T27N), and Rab11 (EGFP-Rab11S25N). The spent media was replaced with fresh media after 12 hrs of nucleofection. The cells were incubated for additional 36 hrs under normoxic (8% O_2_) or hypoxic (2% O_2_) culture conditions before analyzing sFlt1 mRNA and protein levels. ***A*** **:** Quantitative RT-PCR analysis showing *sFlt1* transcript levels relative to normoxia control. Data  =  mean ± SEM (n = 3). ***B*** **:** Spent culture media were prepared for immunoprecipitation with Flt1 Ab. Blot shows relative levels of secreted sFlt1 in the culture medium. ***C*** **:** Band densities from experiments as in ***B*** were quantified by densitometric analysis. ***D*** **:** After collecting the spent culture media for analysis, the remaining cells were lysed and a relative level of sFlt1 was determined by western blot (WB) analysis. E: Experiment was performed as in ***B*** **;** amount of sFlt1 in spent culture supernatant was measured by sandwich ELISA. Values in ***C*** **,** ***E*** are expressed as mean ± SEM (n = 70 cells for each condition, from 3 separate experiments).

JEG3 cells were nucleofected with plasmids expressing dominant-negative forms of Arf1, Arf6, and Rab11. As expected, our data show that hypoxia increases the *sFlt1* mRNA expression and secretion of sFlt1 (Figure 4A, B, C). Expression of mutant forms of Rab11, Arf1, and Arf6 in JEG3 cells did not alter the hypoxia-induced increased expression of *sFlt1* mRNA (Figure S3, Figure 4A), but resulted in a 2–3 fold reduction in the amount of sFlt1 secreted in the medium relative to corresponding mock (Figure 4B,C), and increased its intracellular accumulation during both normoxic (8% O_2_) and hypoxic (2% O_2_) culture conditions (Figure 4D). We further examined the effect of Arf1, Arf6, and Rab11 dominant-negative mutants on the concentration of sFlt1 secreted into the extracellular medium during normoxia and hypoxia via ELISA. The secreted sFlt1 protein levels during normoxic culture (3.5 ng/ml/5×10^5^ cells) decreased approximately 30–37% upon expression of mutant forms of Arf1, Arf6, and Rab11. During hypoxic conditions, the level of sFlt1 was increased (18.6 ng/ml/5×10^5^ cells) relative to normoxic conditions, as was expected. The expression of the dominant-negative GTPases under hypoxic conditions decreased the sFlt1 concentration in the medium to a similar degree (30–35%) (Figure 4E).

### Role of Arf1, Arf6, and Rab11 on VEGF-A-induced cell proliferation and cell migration

Because sFlt1 can trap VEGF-A in the extracellular space and thus inhibit endothelial cell growth and migration [3], we next examined the effect of dominant negative mutant forms of Arf1, Arf6, and Rab11 on VEGF-induced EC-sFlt1 cell proliferation and migration.

**Figure 5:**
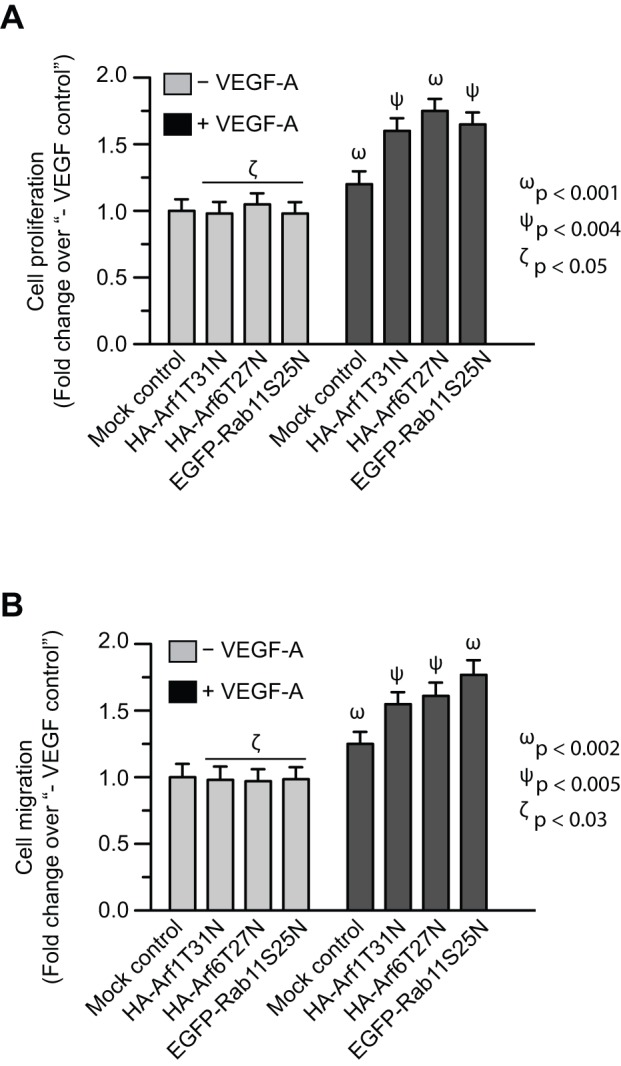
Inhibition of Rab11, Arf1, and Arf6 function enhances biological activity of VEGF-A as revealed by cell proliferation and migration assays. ***A*, *B*:** EC-sFlt1 cells were either mock treated (control) or were nucleofected with dominant-negative forms of Arf1 (HA-tagged-Arf1T31N), Arf6 (HA-tagged-Arf6T27N), and Rab11 (EGFP-Rab11S25N). Cells were harvested at 36 hrs post-transfection for subsequent assays. ***A*** **:** Samples were serum-starved and then treated with VEGF-A (5 ng/ml) for 24 hrs. Cell proliferation assays were carried out using the MTT assay. Cell proliferation values were normalized to that of unstimulated, mock-transfected controls. ***B*** **:** Directional migration of cells toward VEGF-A (5 ng/ml) by Boyden chamber assay, with VEGF present in the lower well. The number of migrating cells was normalized to that in unstimulated, mock-transfected controls. Values represent mean ± SEM (n = 3).

VEGF-A (5 ng/ml) moderately, but significantly, stimulated the proliferation of cells by 1.2 fold. However, expression of Arf1, Arf6, and Rab11 mutant forms increased effect of VEGF-induced cell proliferation by 1.6–1.75 fold (Figure 5A). The effect of these mutants on VEGF-induced cell migration was similar to the effect observed with cell proliferation assay (Figure 5B). These results indicate that functional inhibition of Arf1, Arf6, and Rab11 reduces sFlt1 secreted by the EC-sFlt1 cells and further stimulated VEGF-induced EC proliferation and migration probably in an autocrine fashion.

## Discussion

**Figure 6:**
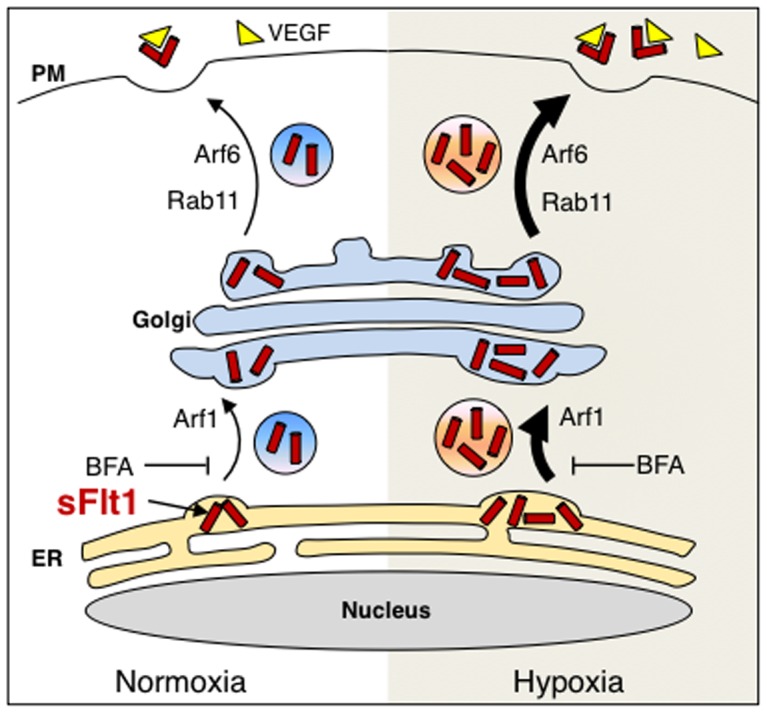
Schematic diagram of sFlt1 secretion and its secretory transport itinerary. A tentative model summarizing our current view. After its initial synthesis, sFlt1 is transported from the ER to the Golgi complex; this step is sensitive to BFA treatment and also requires Arf1 GTPase. Soluble Flt1 (sFlt1) transport from the Golgi complex for subsequent secretion requires Rab11 and Arf6 GTPases. Our data predict that sFlt1 secretion is dependent on Arf1-, Arf6-, and Rab11-regulated intracellular vesicular trafficking events along the secretory pathway.

Soluble Flt1 is an alternatively spliced gene product of Flt1, which has been implicated in pathologies associated with preeclampsia and also during physiological functions such as blood vessel sprouting. It is secreted from endothelial cells into their immediate extracellular space as well as into the general circulation and reduces the bioavailability of VEGF by binding and sequestering this growth factor [43], [44]. Increased secretion may contribute to higher blood levels as seen in disease states such as preeclampsia. sFlt1 is significantly increased in maternal circulation prior to the onset of clinical preeclampsia [45], [46]. To date, the mechanism of secretory transport leading to sFlt1 secretion is not known. Better understanding of secretory transport pathway and the regulatory proteins involved could be used to generate potential therapeutic targets to modulate secretion of sFlt1. In this study, we demonstrate that secretory transport of sFlt1 through the Golgi complex is prerequisite for its secretion. Our results support a model (outlined in Figure 6) where we provide evidence that the small GTPases Rab11, Arf1, and Arf6 are involved in the secretory transport of sFlt1. Inhibition of their function during normoxic and hypoxic conditions blocks sFlt1 secretion with concomitant intracellular accumulation.

After initial synthesis, sFlt1 could be detected in the extracellular medium after 4 hrs which suggests sFlt1 may be transported via vesicular trafficking through the various secretory organelles such as the ER, Golgi, and post-Golgi compartments on its way to secretion into the extracellular medium. Cargo capture is usually presented as the main mechanism for the selective transport of newly synthesized proteins from the ER to the Golgi complex [47], [48], [49]. A group of membrane proteins, including vesicular stomatitis virus glycoprotein (VSVG), have a sequence element (DXE) in their cytosolic domain that directly interacts with proteins of the COPII coat and helps to accumulate these proteins in ER exit sites and accelerates their transport to the Golgi complex [50]. Unlike full-length Flt1, which contains a transmembrane and cytosolic domain, sFlt1 is soluble and contains only the ligand-binding domain; as such, it is most likely packaged within the lumen of the vesicle, which transports it from the ER to the Golgi complex. Soluble proteins have short half time of secretion, which is approximately 50 min for proteins such as albumin and alpha1-antitripsin [51], [52]. However, despite being a soluble vesicle luminal protein, sFlt1 has a relatively long half time for secretion of 4 hrs. The difference is most likely explained by the fact that the reported transport rates include the time taken required folding and glycosylation in the Golgi, which may be more extensive since sFlt1 is a heavily glycosylated protein [19], [53].

The ADP-ribosylation factor (Arf) family of proteins belongs to the Ras superfamily of small GTPases and regulates vesicular traffic by recruiting coat proteins at membrane surfaces [54]. Arf1 and Arf6 are involved in the secretory transport of cargo molecules during various trafficking events such as endocytosis and phagocytosis. Cytosolic coat protein, COPI, recruitment to membranes appears to be essential for the biogenesis of the Golgi complex and for secretory trafficking [55]. Prevention of COPI recruitment by expressing inactive forms of the ADP-ribosylation factor (Arf) causes the collapse of the Golgi into the endoplasmic reticulum (ER) and arrests trafficking of soluble and transmembrane proteins at the ER [56]. Over-expressing a mutant Arf1 (Arf1-T31N) causes dissociation of COPI from membranes [31]. Here, we show that loss of Arf1 function causes intracellular accumulation of sFlt1 and blocks its secretion probably by interfering with COPI recruitment to membranes and blocking sFlt1 trafficking between the ER and the Golgi complex.

Arf6 is the least-conserved member of the Arf family of proteins and shares 66% amino-acid identity with Arf1, but unlike Arf1, Arf6 has no effect on Golgi membrane dynamics. Instead, it localizes to the plasma membrane and endosomal compartments, where it regulates endocytic membrane recycling [54]. Arf6 has also been shown to have a role in secretion in certain cell types [33], [57]. Expression of the constitutively inactive Arf6 (T27N) mutant inhibits secretagogue-dependent exocytosis from PC12 cells [33]. Our results demonstrate that an Arf6 mutant blocks secretion of sFlt1 suggesting role of Arf6 in sFlt1 secretion. Arf6's role in the exocytic process is functionally linked with phospholipase D (PLD)1 as mutant Arf6 impaired PLD1 stimulation. Future studies will be needed to establish if indeed PLD1 activation is needed for sFlt1 secretion. In many respects, Arf6 regulation of exocytotic processes is similar to the Arf6-induced recycling of endosomal membrane.

Rab11 is a member of the Rab family of proteins; it is localized to the endocytotic recycling endosomes and the trans-Golgi network and is involved in the endosomal recycling pathway in mammalian cells [58], [59], [60]. Rab11 has also been found to regulate the secretory process during both regulated and constitutive secretion in the PC12 cell line [28], [61]. Our results show that secretion of sFlt1 requires functional Rab11, since expression of a dominant-negative Rab11 blocked the secretion of sFlt1. Since Rab11 is also known to regulate endocytic recycling [60], it will be interesting to determine if sFlt1 co-traffics along with endocytic recycling cargo or is segregated from it.

The massive sFlt1 production observed in clinical preeclampsia is believed to be secondary to placental hypoxia [5], [62]. However, it is still unclear whether placental hypoxia or excess sFlt1 production is the trigger event in the pathogenesis of preeclampsia. Nevertheless, our results show that sFlt1 secretion during both normoxic and hypoxic conditions requires functional Arf1, Arf6, and Rab11 suggesting that sFlt1's trafficking itinerary remains unaltered during pathological states. We believe a comprehensive understanding of sFlt1 trafficking and secretion mechanisms may provide insight into new targets or new strategies for reducing or controlling sFlt1 release in preeclamptic disease.

## Materials and Methods

### Reagents

The goat polyclonal antibody (Ab) against human VEGFR1 (Flt1) was purchased from R&D Systems. The rabbit polyclonal Ab against human trans-Golgi network 46 (TGN46) was purchased from AbD Serotec. The rabbit polyclonal Ab against HA epitope was obtained from Novus Biologicals. The mouse monoclonal Ab against green fluorescence protein (GFP) was obtained from UCDavis/NIH NeuroMab Facility. Abs against Arf1, Arf6, and Rab11 were from Abcam (ab108347, Cambridge, MA), Thermo Scientific (PA1-093X, Rockford, IL), and Millipore (05-853, Billerica, MA), respectively. Fugene HD transfection reagent, protease inhibitor, and phosphatase inhibitor cocktail tablets were obtained from Roche Diagnostics. Alexa Fluor-conjugated secondary antibodies and the mouse monoclonal Ab against v5 epitope were obtained from Invitrogen, Molecular Probes. Vectashield mounting medium was obtained from Vector Laboratories. Brefeldin A (BFA) and the monoclonal Ab against α-tubulin were purchased from Sigma-Aldrich. Protein A&G sepharose beads and Amplify-fluorographic Reagent were purchased from GE Healthcare. SuperSignal West Femto ECL reagent was obtained from Thermo Scientific, USA. ^35^S-methionine and cysteine (EasyTag Express ^35^S Protein labeling mix) was obtained from PerkinElmer (Waltham, MA). Plasmid construct of sFlt1 was cloned in pcDNA3.1 vector, which contains a C-terminal v5-epitope tag as described before [19]. Recombinant adenoviruses expressing the cytosolic domains of syntaxin 6 and syntaxin 16 (designated syntaxin 6-cyto, syntaxin 16-cyto) and plasmid form of syntaxin 4-cyto were used as described previously ^18, 24^. HA-tagged Arf1 (T31N) and Arf6 (T27N) plasmids are dominant negative mutant forms of Arf1 and Arf6 respectively, were gift from Dr. Harish Radhakrishna (Georgia Institute of Technology, Atlanta, GA), and has been described before [63]. Plasmid constructs EGFP-Rab5aN133I and EGFP-Rab11S25N is dominant negative mutants forms of Rab5a and Rab11a respectively, and were generated by us as described before [60], [64]. Plasmid constructs for dominant mutant forms of Rab6 (myc-tagged Rab6T27N in pcDNA3.1) and Rab8 (HA-tagged Rab8T22N in pcDNA3.1) were obtained from Dr. Richard Pagano (Mayo Clinic, Rochester, MN). siRNAs against Arf1 (5′-AACATCTTCGCCAACCTCTTC-3′), Arf6 (5′-AAGGTCTCATCTTCGTAGTGG-3′), Rab11 (5′-AATGTCAGACAGACGCGAAAA-3′), and scramble oligonucleotides were obtained from Thermo Scientific Dharmacon (Lafayette, CO).

### Cell culture

Primary human umbilical vein endothelial cells (HUVECs) were obtained from Lonza (Walkersville, MD) and cultured on collagen-coated plates in complete medium (endothelial-cell basal medium containing supplements supplied by Lonza). To generate HUVECs stably expressing sFlt1, passage 3 cells were transfected with plasmids encoding v5-tagged sFlt1 constructs via nucleofection using an Amaxa nucleoporator. Transfected cells were then selected in neomycin G418 (500 μg/ml) and clones exhibiting both high levels of v5-epitope and Flt1 Ab reactivity were selected and labeled EC-sFlt1 cells. In subsequent experiments, EC-sFlt1 stable clones were utilized between passages 5 and 7. The human choriocarcinoma cell line, JEG-3, was purchased from the American Type Culture Collection (ATCC) and cultured in Dulbecco's modified Eagle's medium (DMEM) containing 10% fetal bovine serum (FBS).

To study the roles of various dominant-mutant forms or siRNAs against small GTPases on sFlt1 secretion, transfected EC-sFlt1 or JEG-3 cells were grown to 70–80% confluency. Next, in order to create a hypoxic environment, cells were incubated at 37°C in a Heraeus Heracell 150 tri-gas cell culture incubator (Thermo Fisher Scientific, Rockford, IL) with 2% O_2_ and 5% CO_2_ for 48 hrs. In parallel, control dishes were cultured under standard culture conditions (8% O_2_ and 5% CO_2_).

### Western blotting and Immunoprecipitation

For western blotting (WB) assays, cells were washed twice with ice-cold PBS and harvested with gentle scraping. The cells were then lysed in RIPA buffer (50 mM Tris, pH 7.4, 150 mM NaCl, 1% Nonidet P-40, 0.1% SDS, 0.5% sodium deoxycholate) supplemented with mixture of proteases and phosphatases inhibitors. After centrifugation at 3000 g for 5 min, supernatants were collected for whole cell lysate. Cultured medium was also collected before washing step and centrifuged at 300 g for 5 min to discard precipitation of dead cell portion. Proteins in both whole cell lysate as well as medium were resolved on SDS-PAGE followed by IB and visualization of Ab binding with the ECL reagent. For immunoprecipitation (IP) assays, whole cell lysate and medium were precleared with uncoupled protein G beads and incubated overnight with Ab-coupled beads in rotator at 4°C. Immune complexes were washed with lysis buffer and then subjected to SDS-PAGE and IB. All of protein intensities were measured using Image J software Version 1.44b (NIH).

### ^35^S-metabolic labeling

HUVECs were starved for 1 hr with DMEM Met-Cys-free medium supplemented with 5% dialyzed FBS. Met-Cys-starved cells were pulsed with 250 μCi/mL of both ^35^S-methionine and cysteine (^35^S-Met/Cys) for 20 min at 37°C. After incubation of labeled cells in chase medium (10% dialyzed FBS and a 100-fold excess of methionine and cysteine) for indicated chase periods, cells were lysed in RIPA buffer. sFlt1 was immunoprecipitated using a goat polyclonal Ab against Flt1 and protein G sepharose beads. Then the samples were resolved by SDS-PAGE. Gels were subsequently fixed for 30 min in fixing solution (isopropanol:water:acetic acid, 25∶65∶10) and treated with Amplify-fluorographic Reagent for 30 min before subjected to autoradiopraphy. The autoradiographic signals from films were measured using Image J software. For treatment of BFA, HUVECs were treated with 1 μg/mL of BFA for 6 hrs from 1-hr metabolic labeling period to the end of 5-hrs chase period.

### Immunofluorescence

For immunofluorescence (IF) assays, cells were grown on acid-washed glass coverslips in 35 mm culture dishes. Cells were treated with 1 μg/mL of BFA for indicated time before fixation. Cells were fixed in 4% paraformaldehyde (PFA) in PBS for 25 min at room temperature (RT), quenched with 100 mM glycine in PBS for 10 min at RT, and washed twice with PBS. Cells were then permeabilized with 0.1% Triton X-100 in PBS for 5 min at RT, blocked with PBS containing 5% glycine and 5% normal goat or donkey serum for 1 hr, and incubated overnight at 4°C with primary Abs. The next day, coverslips containing cells were incubated for 1 hr in a 1∶200 dilution of either Alexa Fluor 488- or Alexa Fluor 594-conjugated secondary Ab and mounted using Vecta Shield mounting medium containing DAPI. Fluorescence images were acquired using a Leica spinning-disk confocal microscope equipped with a Hamamatsu EM-CCD digital camera (Hamamatsu Photonics) and the Metamorph software (Molecular Devices Corporation). All images were acquired using a 63x, 1.3 NA objective. The same exposure time and fluorescence power were applied to the same batch of experiments.

### sVEGFR1/sFlt1 detection by ELISA

The concentration of secreted sFlt1 protein in spent culture supernatant was determined quantitatively with a Quantikine ELISA kit (R&D Systems) based on sandwich immunoassay technique. The media samples were diluted and added to the 96-well plate coated with a monoclonal Ab specific for VEGFR1/sFlt1 and were allowed to incubate for 2 hr. Any unbound sFlt1 was removed by washing with a buffer and then incubatedwith an enzyme-linked polyclonal Ab specific for VEGFR1/sFlt1. The excess of Ab-enzyme conjugate was washed off and the samples were then incubated with a substratesolution. Enzyme-catalyzed chromogen formation was quantified by measuring the visible absorbance at 450 nm. Using a calibration curve plotted with recombinant human sFlt1, the concentrations of expressed sFlt1 (in pg/ml of spent culture supernatant from 5×10^5^ cells) were calculated from the absorbance value following nucleofection-mediated cell transfection with various plasmids and cultured under normoxic and hypoxic conditions.

### qPCR (quantitative PCR)

EC-sFlt1 were either mock transfected or transfected with plasmid constructs expressing dominant negative mutant forms of Arf1, Arf6, or Rab11 and cultured for 36–48 hours in hypoxic or normoxic conditions. Total RNA was isolated using a Qiagen RNeasy kit (Qiagen) and quantified. cDNA was synthesized from total RNA, using the iScript cDNA synthesis kit (Bio-Rad Laboratories). Thermocycler conditions included a 5 min incubation at 25°C, a 30 min incubation at 42°C, and a 5 min incubation at 85°C. The cDNAs were subjected to qPCR analysis with the following 5′→3′ primers (sense and antisense respectively): sFlt1, ACA ATC AGA GGT GAG CAC TGC AA and TCC GAG CCT GAA AGT TAG CAA, amplicon length 180 bp; human 18S rRNA (house-keeping gene) CCTTGGATGTGGTAGCCGTTT and AACTTTCGATGGTAGTCGCCG, amplicon length 105 bp. Primers were designed using the Primer Express software (Applied Biosystems). The assay was performed in a 96-well optical plate, with a final reaction volume of 20 *μ*l, including synthesized cDNA (20 ng), oligonucleotide primers (100 *μ*M each), and 2× SYBR Green/ROX PCR master mix (Bio-Rad Laboratories). Samples were run on an ABI PRISM Sequence Detection System (model 7000, Applied Biosystems). PCR conditions were, one cycle for 10 min at 95°C followed by 30–45 cycles (15–25 cycles for 18S rRNA) of 30 sec denaturation at 95°C, 1 min annealing at 55–60°C, and 1 min extension at 72°C. Samples were checked for non-specific products or primer/dimer amplification by melting curve analysis. The threshold cycle (*C*T) values for the sFlt1 and 18S in all of the samples (analyzed in triplicate) were normalized on the basis of the abundance of the 18S transcript. Relative expression is expressed as 2−d*C*t, where d*C*t  =  (cycle threshold for the sFlt1) – (cycle threshold for the 18S rRNA transcript).

### Cell migration assay

EC-sFlt1 cells were nucleofected with mutant forms of Arf1, Arf6, and Rab11 plasmids and were then used for cell migration measurement as described before [65]. Harvested cells (2×10^4^) were replated in triplicates onto the upper chamber of a Transwell filter with 8 μm pores (6.5 mm, polycarbonate, Costar), and serum starved for 16 hrs. Serum-free medium containing VEGF-A (5 ng/ml) was added to the lower chamber to stimulate chemotaxis. After 4–6 h, cells were fixed with 4% paraformaldehyde in PBS. Non-migrated cells on the upper side of the filter were removed with a cotton swab, and cells on the underside of the filter were stained with 2% crystal violet in 10% ethanol. After extensive washing in water to remove excess crystal violet, filters were dried overnight. Number of migrated cells was determined by eluting crystal violet dye from the stained cells on the underside of the filter in 250 μl of 10% acetic acid (for 15 min) followed by measuring OD at 562 nm.

### Cell proliferation assay

EC-sFlt1 cells were nucleofected with mutant forms of Arf1, Arf6, and Rab11 plasmids and were then used for cell proliferation measurement as described before [66]. After 24 hrs, cells (2×10^4^; untreated or nucleofected ones) were seeded in 96-well plates. After 6 hrs of seeding, cells were serum (0.1%)-starved for 6 hrs and then treated with VEGF-A at 5 ng/ml for 24 hrs. Proliferation was measured using the thiazolyl blue tetrazolium bromide (MTT) colorimetric assay according to the manufacturer's recommendations (Promega). The absorbance at 490 nm was determined using Spectra Fluor PLUS (Molecular Devices, Sunnyvale, CA). For each group, samples were prepared in triplicate. The data are presented as fold increase over levels in controls.

### Statistical analysis

All values are expressed as means ± SEM. Statistical significance was determined using two-sided Student's *t*-test and one-way ANOVA test, using GraphPad Prism version 4.0 software (GraphPad Software, San Diego, CA), and a value of *P*<0.05 was considered significant unless stated otherwise.

## Supporting Information

Figure S1 **Representative image showing extent of plasmid (pEGFP-C1) transfection efficiency in EC-sFlt1 cells by nucleofection.**
(EPS)

Figure S2 ***A*** **: As described in** **Figure 3C** **, EC-sFlt1 cells were nucleofected with plasmid constructs expressing dominant-negative forms of Arf6 (HA-tagged-Arf6T27N), Arf1 (HA-tagged-Arf1T31N), and Rab11 (EGFP-Rab11S25N).** Relative expression of various plasmid constructs in cell lysates were determined by western blotting (WB) with antibodies (Ab) against epitope tags. ***B*** **:** Cell lysates from siRNA treated samples corresponding to data shown in Figure 3C was used for western blotting to determine extent of Arf1, Arf6, and Rab11 knockdown. Band densities from three independent experiments were quantified by densitometric analysis. Values were calculated as the ratio of desitometric band intensity numbers of Arf6, Arf1, or Rab11 to tubulin; this ratio was set at 1 for mock-transfected control EC-sFlt1 cells. Data  =  mean ± SEM.
(EPS)

Figure S3 **As described in** **Figure 4** **, JEG-3 cells were nucleofected with indicated plasmid constructs and subsequently cultured under normoxic and hypoxic culture conditions.** After collecting the spent culture media for analysis, the remaining cells were lysed and expression of various plasmids were determined by western blot (WB) analysis.
(EPS)
