# Correction: Secretion of Soluble Vascular Endothelial Growth Factor Receptor 1 (sVEGFR1/sFlt1) Requires Arf1, Arf6, and Rab11 GTPases

**DOI:** 10.1371/annotation/19ed28f9-9a01-4af9-857d-3ec4aaeceea1

**Published:** 2012-11-20

**Authors:** Jae-Joon Jung, Ajit Tiwari, Shivangi M. Inamdar, Christie P. Thomas, Apollina Goel, Amit Choudhury

There was an error in the funding statement. The correct funding statement is: This work was supported in part by grants from National Institutes of Health, National Heart, Lung, and Blood Institute R01HL089599 to A.C., R01DK090053 to C.T., and R01CA127958 to A.G. The funders had no role in study design, data collection and analysis, decision to publish, or preparation of the manuscript. 

